# Predictive Value of XPD Polymorphisms on Platinum-Based Chemotherapy in Non-Small Cell Lung Cancer: A Systematic Review and Meta-Analysis

**DOI:** 10.1371/journal.pone.0072251

**Published:** 2013-08-19

**Authors:** Mantang Qiu, Xin Yang, Jingwen Hu, Xiangxiang Ding, Feng Jiang, Rong Yin, Lin Xu

**Affiliations:** 1 Department of Thoracic Surgery, Nanjing Medical University Affiliated Cancer Institute of Jiangsu Province, Nanjing, China; 2 The Fourth Clinical College of Nanjing Medical University, Nanjing, China; 3 The First Clinical College of Nanjing Medical University, Nanjing, China; MOE Key Laboratory of Environment and Health, School of Public Health, Tongji Medical College, Huazhong University of Science and Technology, China

## Abstract

**Background:**

The correlation between xeroderma pigmentosum group D (XPD) polymorphisms (Lys751Gln and Asp312Asn) and clinical outcomes of non-small cell lung cancer (NSCLC) patients, who received platinum-based chemotherapy (Pt-chemotherapy), is still inconclusive. This meta-analysis was aimed to systematically review published evidence and ascertain the exact role of XPD polymorphisms.

**Methods:**

Databases of MEDLINE and EMBASE were searched up to April 2013 to identify eligible studies. A rigorous quality assessment of eligible studies was conducted according the Newcastle-Ottawa Quality Assessment Scales. The relationship between XPD polymorphisms and response to Pt-chemotherapy and survival was analyzed.

**Results:**

A total of 22 eligible studies were included and analyzed in this meta-analysis. The overall analysis suggested that the XPD Lys751Gln polymorphism was not associated with response to Pt-chemotherapy or survival. However, the XPD 312Asn allele was significantly associated with poor response to Pt-chemotherapy compared with the Asp312 allele (Asn vs. Asp: OR = 0.435, 95% CI: 0.261–0.726). Additionally, the variant genotype of XPD Asp312Asn polymorphism was associated with favorable survival in Caucasian (AspAsn vs. AspAsp: HR = 0.781, 95% CI: 0.619–0.986) but unfavorable survival in Asian (AspAsn+AsnAsn vs. AspAsp: HR = 1.550, 95% CI: 1.038–2.315).

**Conclusions:**

These results suggest that XPD Asp312Asn polymorphism may function as a predictive biomarker on platinum-based chemotherapy in NSCLC and further studies are warranted.

## Introduction

Lung cancer is the leading cause of cancer related death worldwide [1]. Non-small cell lung cancer (NSCLC) accounts for about 80% of primary lung cancers, most of which were diagnosed at the advanced stage [2]. Chemotherapy is the main treatment of choice for advanced NSCLC [3], [4]. Among various types of chemotherapy regimens, platinum-based chemotherapy (Pt-CP) improves survival and has been the standard chemotherapy for years [5], [6]. However, the 5-year survival rate of NSCLC remains less than 15% [7], and the efficacy of platinum-based chemotherapy varies among individuals, with a response rate of 26–60% [8]. To optimize individualized chemotherapy, a predictive biomarker is needed to identify those who are susceptible to Pt-chemotherapy.

The cytotoxicity effect of platinum compounds, such as cisplatin and carboplatin, is due to the formation of platinum-DNA adducts that leads to bulky distortion of DNA, destabilization of the double helix, inhibition of DNA replication, transcription and ultimately death of tumor cells. It has been hypothesized that suboptimal DNA repair capacity may lead to decreasing removal of platinum-DNA adducts, and eventually favorable clinical outcomes [9], [10].

Nucleotide excision repair (NER) is the major pathway for the removal of bulky DNA adducts [11], [12]. It has been demonstrated that over-expression of xeroderma pigmentosum group D (XPD, also named excision repair cross-complementing group 2, ERCC2), which is a key member of the multistep NER pathway, is associated with cisplatin resistance [13]. There are two extensively investigated non-synonymous single nucleotide polymorphisms (SNPs) in the coding region of the XPD gene: Lys751Gln (rs13181, G>A) and Asp312Asn (rs1799793, A>C). Clinical studies suggested that the XPD Lys751Gln and Asp312Asn polymorphisms might predict response to Pt-CP and survival of patients with NSCLC [14]–[17]. However, previous meta-analyses suggested no association of XPD polymorphisms with clinical outcomes in NSCLC [18]–[20]. However, significant associations were observed in recent studies and an update meta-analysis is necessary [14], [15], [17]. For example, in a recent observational study of 353 NSCLC patients (III-IV) receiving Pt-CP, Wu and colleagues found that the Asp312Asn and Lys751Gln polymorphisms were significantly associated with poor survival [18].

Thus, by identifying all eligible studies, we performed this meta-analysis to re-evaluate the relationship between XPD polymorphisms (Lys751Gln and Asp312Asn) and clinical outcome (response and overall survival) in NSCLC patients treated with Pt-CP.

## Materials and Methods

### Data Sources and Searching Strategy

This meta-analysis was conducted and reported in accordance with the PRISMA guidelines for systematic reviews and meta-analyses (Table S1. PRISMA checklist) [21]. A comprehensive search of PubMed and EMBASE databases was carried out up to April 2012 to identify published studies that investigated the relationship between XPD polymorphisms and clinical outcomes of NSCLC patients treated with Pt-CP. Medical subheadings and key words such as “lung cancer” or “lung neoplasm” and “XPD” or “xeroderma pigmentosum group D” and “polymorphism” or “variation” were used for database searching. Other alternative spellings were also considered. References lists of related review articles and original eligible studies were manually searched to identify studies missed by the database search.

### Study Identification and Inclusion Criteria

Records identified from databases were primarily screened by titles and abstracts, and then full-text articles were retrieved for further assess the eligibility. Studies met the following criteria were included: 1) NSCLC patients; 2) patients treated with Pt-CP; 3) reporting relationship between XPD polymorphisms and response or survival; 4) available data for quantitative synthesis, namely genotype distribution data for response and hazard ratio (HR) and 95% confidence intervals (CIs) for overall survival (OS). Studies without available data were excluded. For multi-reports from the same center, only the most recent one was included. All searching records were screened by two authors (Qiu and Yang), with discrepancies solved by discussion with another author (Yin).

### Outcomes Definition

Response to Pt-chemotherapy and overall survival were the primary outcomes in this meta-analysis. Response to chemotherapy was assessed with RECIST criteria [22] or WHO criteria, namely, “good response” was defined as “complete response+partial response” and “poor response” was “stable disease+progressive disease”. Data of overall survival (HR and 95% CIs) were extracted from studies directly according to studies’ own definition.

### Data Extraction

Data of eligible studies were extracted by two authors (Qiu and Yang) independently in duplicate with a pre-designed data collection form. The two authors reached consensus on each item. The following data was collected, name of first author, year of publication, country, ethnicity, chemotherapy regimens,number of patients, TNM stages, age, percentage of male, SNPs investigated, genotype distribution data among responders and non-responders and HR and corresponding 95% CIs of OS. For OS, we collected HR and CIs of each comparison. Ethnicity descents were simply categorized as Asian or Caucasian. The two authors reached consensus on each item.

### Quality Assessment

Methodological quality of included studies was assessed with the Newcastle-Ottawa scale (NOS) for cohort studies [23], which evaluates 3 aspects of a cohort study: selection, comparability and outcome. The NOS identifies high quality with a star and there are a maximum of 4 stars, 2 stars and 3 stars in the “selection”, “comparability” and ”outcome”, respectively. Also, quality assessment was performed by two authors (Yang and Hu) independently.

### Statistical Analysis

Pooled odds ratio and 95% CIs were calculated to estimate the association strength of XPD polymorphisms with overall response rate. The 95% CIs were utilized for statistical significance test and a 95% CI without 1 suggested significant difference. For ORR, 5 genetic comparison models were analyzed (A: allele comparison, A vs. a; B: heterozygote comparison, Aa vs. aa; C: homozygote comparison, AA vs. aa; D: dominant model, AA+Aa vs. aa; E: recessive model, AA vs. Aa+aa; A, variant allele; a, wild allele; the 751Gln and 312Asn alleles were considered as variant alleles). The genotype distribution data was directly used to estimate the pooled ORs and 95% CIs of ORR. For OS, HRs and CIs retrieved from each study were calculated to estimate the pooled HRs and 95% CIs. Also, the 95% CIs pooled HRs were used for statistical test. Pooled HRs for homozygote comparison, heterozygote comparison and dominant model were calculated.

Heterogeneity was measured by chi-square based Q test, and p<0.10 indicated the existence of significant heterogeneity [24]. The fixed-effects model and random-effects model were utilized to pool data from eligible studies. The fixed-effects model was used in the absence of significant heterogeneity; otherwise, the random-effects model was applied. Sub-group analyses according to ethnicities. Since gemcitabine-platinum (GP) chemotherapy was reported in many studies, we also performed a sub-group analysis for studies with GP chemotherapy. Begg’s funnel plot and Egger’s linear regression test were conducted to detect publication bias, and a p<0.05 was considered significant [25].

All statistical analyses were carried out with STATA software (version 10.0, StataCorp, College Station, Texas USA). All p values are two-side.

## Results

### Study Selection

**Figure 1:**
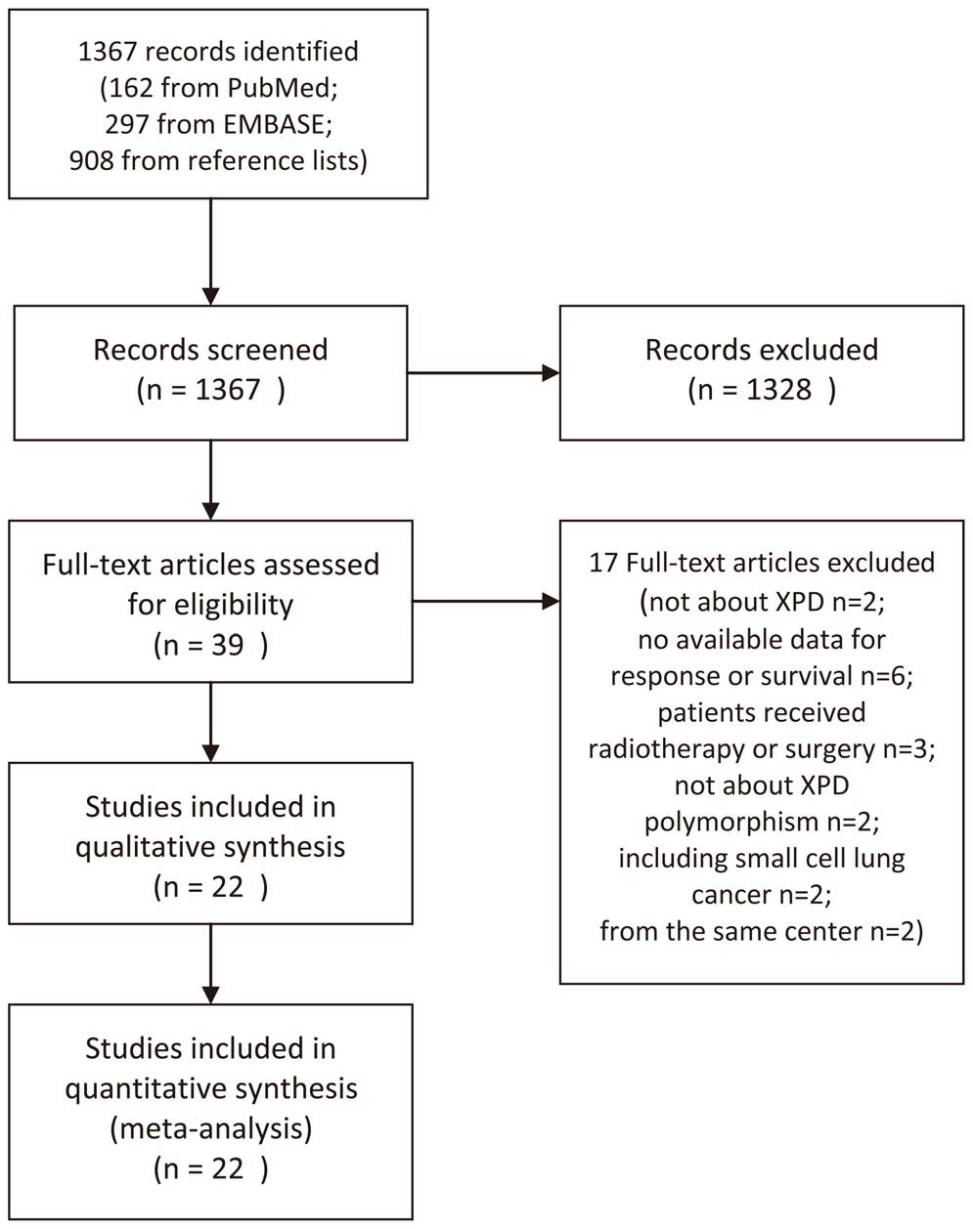
Flow Diagram.

Overall, 1367 records were identified by a primary search of databases and reference lists. After screening of titles and abstracts, a total of 39 full-text articles were retrieved for further evaluate the eligibility. Finally, 22 eligible studies were identified and included in this meta-analysis according to our inclusion and exclusion criteria [10], [14]–[17], [26]–[42]. The process of study selection was shown in Figure 1.

### Overview of Eligible Studies

Baseline characteristics of eligible studies were shown in Table 1. A total of 4383 NSCLC patients were included. The sample size ranged from 62 to 632. 10 studies were conducted among Caucasian population and 12 studies were among Asian population. For studies about Caucasian population, they were conducted in Spain, Italy, Netherlands, USA and Greece. All studies enrolled patients with advanced stage (IIIA-IV or IIIB-IV), except one study by Zhang (I-IV) [14]. Platinum-based doublet chemotherapy was reported in all studies and GP chemotherapy was the most popular regimen. A total of 16 studies reported the correlation between XPD polymorphisms and OS, and 18 studies reported XPD polymorphisms and response. XPD Lys751Gln polymorphism was investigated in 20 studies and Asp312Asn polymorphism were investigated in 12 studies.

**Table 1:** Baseline characteristics of included studies.

Author	Year	Country	Ethnicity	Treatment	Cases	Age	Male	TNMStage	SNPs
Provencio M	2012	Spain	Caucasian	vinorelbine+cisplatin	180	62(39–78)	87%	IIIB–IV	Lys751Gln,Asp312Asn
Zhang ZY	2012	China	Asian	gemcitabine+cisplatin	632	62.6+3.7	76.50%	I–IV	Lys751Gln,Asp312Asn
Tiseo M	2012	Netherlands,Italy	Caucasian	Pemetrexed/+carboplatin	208	60(36–84)	63.00%	IIIB–IV	Lys751Gln
Liao WY*	2012	China	Asian	gemcitabine+platinum	62	57(36–78)	56.50%	IIIB–IV	Lys751Gln,Asp312Asn
Wu W	2012	China	Asian	platinum-basedchemotherapy	353	57(32–80)	69.70%	IIIA–IV	Lys751Gln,Asp312Asn
Chen X	2012	China	Asian	platinum-basedchemotherapy	355	60(32–78)	69.90%	IIIB–IV	Lys751Gln
Ludovini V	2011	Italy	Caucasian	cisplatin+gemcitabine	192	62(25–81)	74.00%	IIIB–IV	Lys751Gln
Joerger M	2012	Netherlands	Caucasian	gemcitabine+platinum	137	59.7(37–79)	56%	IIIB–IV	Lys751Gln,Asp312Asn
Li D	2012	China	Asian	platinum-basedchemotherapy	89	59.08(21–84)	71.90%	IIIA–IV	Lys751Gln
Ren S	2012	China	Asian	platinum-based	340	60(30–78)	68.20%	IIIB–IV	Lys751Gln
Liu L	2011	China	Asian	platinum-based	199	59(29–74)	64.70%	IIIA–IV	Lys751Gln
Viñolas N	2011	Spain	Caucasian	cisplatin+vinorelbine	94	61(37–77)	84%	IIIB–IV	Lys751Gln,Asp312Asn
Li F	2010	China	Asian	platinum-based	115	<60,63	67.80%	IIIB–IV	Lys751Gln
Yao CY	2009	China	Asian	platinum-based	108	61(37–79)	65.70%	IIIB–IV	Lys751Gln
Gandara DR	2009	Japan,Us	Mixed	paclitaxel+carboplatin	526	63(28–81)	58%	IIIB–IV	Lys751Gln
Kalikaki A	2009	Greece	Caucasian	platinum-based	119	61(39–85)	84.80%	IIIA–IV	Lys751Gln,Asp312Asn
Tibaldi C	2008	Italy	Caucasian	gemcitabine+cisplatin	65	65(44–77)	78.50%	IIIB–IV	Lys751Gln,Asp312Asn
Yuan P	2006	China	Asian	platinum-based	200	56(30–74)	65%	IIIB–IV	Lys751Gln
de las Peñas R	2006	Spain	Caucasian	gemcitabine+cisplatin	135	62(31–81)	92%	IIIB–IV	Lys751Gln,Asp312Asn
Isla D	2004	Spain	Caucasian	cisplatin+docetaxel	62	62(35–78)	76.70%	IIIB–IV	Asp312Asn
Gurubhagavatula S	2004	USA	Caucasian	platinum-based	103	58(32–77)	51%	IIIA–IV	Asp312Asn
Ryu JS	2004	Korea	Asian	cisplatin combination	109	60(32–78)	80.70%	IIIB–IV	Lys751Gln,Asp312Asn

Age is presented as median and range;

* data were extracted from the training.

All included studies were assessed with the NOS quality scale, and the detail quality score could be found in (Table S2. Quality assessment of eligible studies with Newcastle-Ottawa Scale). The study reported by Zhang [14], was given only 1 star in “comparability”, which included NSCLC patients with stages of I-IV. In the section of “comparability”, we considered chemotherapy regimens as the most important factor.

### XPD Polymorphisms and Response

#### XPD Lys751Gln polymorphism

Fourteen studies, including 2241 NSCLC patients, were pooled to estimate the association strength of Lys751Gln polymorphism with response. In overall analysis, the Lys751Gln polymorphism was not associated with response rate in any of the 5 comparison models (Table 2). Sub-group analysis by ethnicity showed no differences between Asian and Caucasian; however, in dominant model, carriers of the 751Gln allele might have good response than those of the Lys751 allele in Caucasian population (LysGln+GlnGln vs. LysLys, OR = 1.597, 95% CI:0.994–2.564, P_heterogeneity_ = 0.064). There was no significant association of XPD Lys751Gln polymorphism with response in the sub-group of GP chemotherapy. No evidence of publication bias was detected.

**Table 2:** Meta-analysis results about XPD polymorphisms and response to platinum-based chemotherapy.

	Allele Comparison			Homozygote Comparison			Heterozygote Comparison			Recessive Model			Dominant Model		
	Studies	OR(95% CI)	Phet	Studies	OR(95% CI)	Phet	Studies	OR(95% CI)	Phet	Studies	OR(95% CI)	Phet	Studies	OR(95% CI)	Phet
XPD Lys751Gln (rs13181, A>C)															
Overall	9	1.204(0.753,1.923)	0.001	5	1.688(0.499,5.715)	0.009	10	1.087(0.802,1.473)	0.772	6	1.401(0.595,3.300)	0.019	12	1.283(0.933,1.764)	0.076
Asian	4	0.857(0.533,1.376)	0.398	2	0.760(0.227,2.546)	0.56	5	0.883(0.501,1.555)	0.413	2	0.714(0.223,2.286)	0.661	6	0.989(0.697,1.402)	0.61
Causian	5	1.475(0.792,2.747)	<0.001	3	2.979(0.466,19.067)	0.003	5	1.185(0.825,1.702)	0.817	4	1.892(0.611,5.859)	0.007	6	1.597(0.994,2.564)	0.064
GP	4	1.110(0.822,1.499)	0.483	2	1.235(0.614,2.484)	0.417	4	1.083(0.709,1.657)	0.549	2	1.214(0.658,2.237)	0.427	5	1.336(0.922,1.935)	0.185
XPD Asp312Asn (rs1799793, G>A)															
Overall	7	0.435(0.261,0.726)*	0.017	5	1.328(0.734,2.401)	0.288	7	1.186(0.815,1.726)	0.503	5	1.223(0.711,2.103)	0.24	9	1.185(0.875,1.605)	0.715
Asian	2	0.058(0.014,0.243)*	0.63				2	1.959(0.604,6.349)	0.896				3	1.664(0.860,3.220)	0.934
Causian	5	0.548(0.380,0.791)*	0.194	5	1.328(0.734,2.401)	0.288	5	1.118(0.752,1.663)	0.34	5	1.223(0.711,2.103)	0.24	6	1.089(0.775,1.530)	0.547
GP	3	1.235(0.815,1.870)	0.329	2	1.390(0.610,3.169)	0.227	3	1.346(0.703,2.576)	0.372	2	1.201(0.578,2.496)	0.496	3	1.375(0.750,2.523)	0.302

OR, odds ratio; CI, confidence intervals; Phet, p value of heterogeneity; GP, gemcitabine-platinum based chemotherapy;

* significant difference.

#### XPD Asp312Asn polymorphism

**Figure 2:**
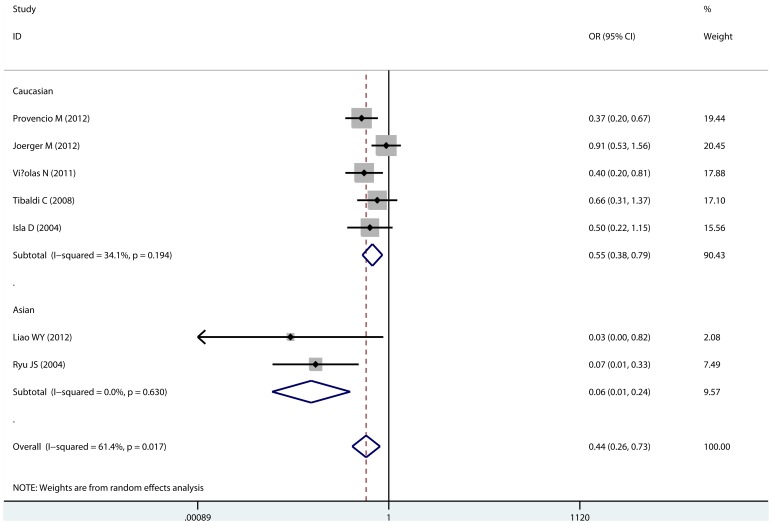
Association of XPD Asp312Asn polymorphism with response to platinum-based chemotherapy. This forest was estimated with allele comparison (Asn vs. Asp).

A total of 9 studies with 1145 individuals were included the analysis for Asp312Asn polymorphism and response. As showed in Table 2, the variant 312Asn allele was associated with poor response compared with the wild Asp312 allele (Asn vs. Asp, OR = 0.435, 95% CI: 0.261–0.726, P_heterogeneity_ = 0.017; Figure 2). The significant association was also observed in the sub-group of Asian population (Asn vs. Asp, OR = 0.058, 95% CI: 0.014–0.243, P_heterogeneity_ = 0.630; Figure 2) and Caucasian population (Asn vs. Asp, OR = 0.548, 95% CI: 0.380–0.791, P_heterogeneity_ = 0.194; Figure 2). No significant association was observed in the sub-group of GP chemotherapy. No publication bias was found.

### XPD Polymorphisms and OS

#### XPD Lys751Gln polymorphism

OS and XPD Lys751Gln polymorphism was reported in 10 studies. By pooling all eligible studies, the Lys751Gln polymorphism was not associated OS in heterozygote comparison (HR = 0.978, 95% CI: 0.803–1.191, P_heterogeneity_ = 0.288) or dominant model (HR = 1.036, 95% CI: 0.786–1.366, P_heterogeneity_ = 0.063). In sub-group analyses, we did not observe any significant association among Asian or Caucasian. The Lys751Gln polymorphism was not associated with OS of patients with GP chemotherapy. No evidence of publication bias was detected.

#### XPD Asp312Asn polymorphism

**Figure 3:**
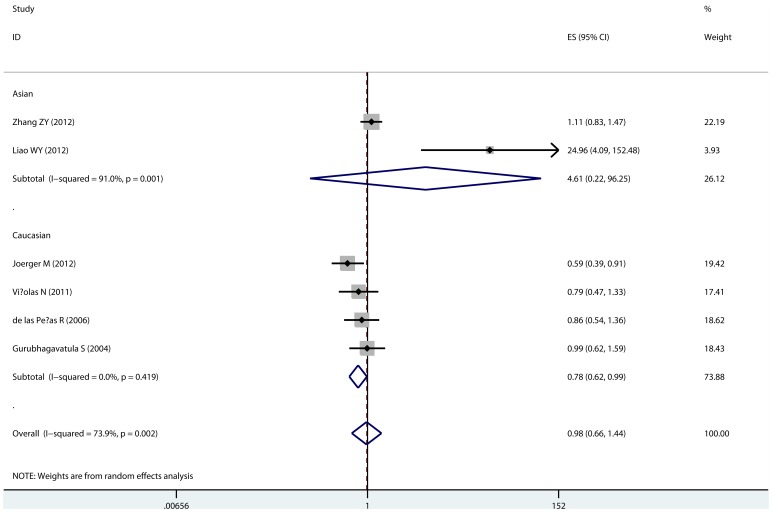
Correlation between XPD Asp312Asn polymorphism and overall survival. This forest was estimated with heterozygote comparison (AspAsn vs. AspAsp).

Seven studies including were available for the analyses of Asp312Asn polymorphism. No significant association of XPD Asp312Asn polymorphism with OS was observed in overall comparison. However, by stratifying analyses according to ethnicity, we found the Asp312Asn genotype was associated with favorable survival in Caucasian population, compared with the wild Asp312Asp genotype (heterozygote comparison, HR = 0.78, 95% CI: 0.619–0.986, P_heterogeneity_ = 0.419; Table 3, Figure 3). While, in the sub-group of Asian, carriers of the 312Asn allele are associated with poor survival (dominant model, HR = 1.550, 95% CI: 1.038–2.315); however, only one study was included in this sub-group. There was no significant association in the sub-group of GP chemotherapy. No evidence of publication bias was detected.

**Table 3:** Meta-analysis results about XPD polymorphisms and overall survival.

	Heterozygote comparison			Homozygote comparison			Dominant model		
	Studies	HR(95% CI)	Phet	Studies	HR(95% CI)	Phet	Studies	HR(95% CI)	Phet
XPD Lys751Gln (rs13181, A>C)									
Overall	7	0.978(0.803,1.191)	0.288	3	0.924(0.562,1.520)	0.091	5	1.036(0.786,1.366)	0.063
Asian	4	1.034(0.723,1.478)	0.152	1	1.340(0.930,1.930)	NA	3	1.167(0.788,1.728)	0.052
Caucasian	3	0.875(0.676,1.131)	0.809	2	0.690(0.431,1.106)	0.835	1	0.700(0.438,1.119)	NA
GP	4	0.984(0.796,1.216)	0.483	2	0.988(0.497,1.964)	0.057			
XPD Asp312Asn (rs1799793, G>A)									
Overall	6	0.976(0.661,1.443)	0.002	7	1.088(0.707,1.672)	0.02	2	1.128(0.590,2.155)	0.037
Asian	2	4.606(0.220,96.246)	0.001	1	1.160(0.808,1.666)	NA	1	1.550(1.038,2.315)*	NA
Caucasian	4	0.781(0.619,0.986)*	0.419	5	1.075(0.580,1.992)	0.01	1	0.800(0.498,1.286)	NA
GP	4	1.125(0.602,2.101)	<0.001	3	0.979(0.577,1.661)	0.036			

HR, hazard ratio; CI, confidence intervals; Phet, p value of heterogeneity; GP, gemcitabine-platinum based chemotherapy;

* significant difference.

## Discussion

In the present meta-analysis, we provide evidence that the XPD Asp312Asn polymorphism could predict poor response in NSCLC patients receiving Pt-chemotherapy, while there was no significant association of Lys751Gln polymorphism with clinical outcomes (response and survival) was observed. The XPD Asp312Asn polymorphism was associated with favorable OS in Caucasian population but unfavorable OS in Asian population.

It has been well documented that platinum agents block DNA replication and lead to tumor cell death by forming DNA-platinum adducts. DNA repair capability is a key factor that modulates sensitivity to platinum. The XPD gene encodes an ATP-dependent 5′–3′ helicase, a subunit of the basal transcription factor IIH (TFIIH) complex that is required for separation of the double helix during NER. Evidence has suggested that XPD overexpression leads to cisplatin resistance [13]. It has been proved that the XPD Lys751Gln and Asp312Asn polymorphisms can modulate NER function, namely the XPD 312Asn and XPD 751Gln are significantly defective in NER [43]. Thus, it is reasonable to conclude that these two functional SNPs may predict sensitivity to Pt-chemotherapy and subsequently clinical outcomes of NSCLC patients with Pt-chemotherapy. Various SNPs are associated with risk and progression of lung cancer [44]–[47] and SNPs are also supposed as potential biomarkers. The functional polymorphism of XPD gene may be a predictive biomarker, given its critical function.

In the present meta-analysis, we showed that the XPD 312Gln allele was associated with poor response to Pt-chemotherapy. Our results were in consistence with a recent meta-analysis [48] about x-ray repair cross-complementing group 1 (XRCC1) polymorphisms and clinical outcomes of Pt-chemotherapy in NSCLC. Both XRCC1 399Gln and XPD 312Asn are associated with defective DNA repair capacity, and also, they both predict unfavorable response to Pt-chemotherapy. In stratified analyses by ethnicity, the XPD 312Asn allele predicted different survival between Asian and Caucasian. However, in the sub-group of Asian population, only 1 study [16] was available for analysis (dominant model), therefore, further studies are needed to validate this association. For XPD Lys751Gln polymorphism, no significant association with response or survival was found and this was consistent with previous meta-analyses [18], [19], although the marginally association was observed in dominant comparison (random-effects model: OR = 1.597, 95% CI: 0.994–2.564; fixed-effects model: OR = 1.629, 95% CI: 1.188–2.235). Compared with two meta-analyses about XPD polymorphism and clinical outcomes of lung cancer, we identified more eligible studies and did comprehensive comparisons. In the present meta-analysis, we included 20 eligible studies for XPD Lys751Gln and 12 studies for Asp312Asn; while for the latest meta-analysis by Yin and colleagues [4], the number of studies included for each SNP was 11 and 7, respectively. Additionally, we performed 5 comparison models for response and 3 models for survival, while only dominant model for response and survival were analyzed in the two meta-analyses [3], [4].

The strength of the present analysis lies in inclusion of 22 eligible studies. To minimize bias, we excluded studies that include patients receiving surgery or radiotherapy. Publication bias was not detected in our meta-analysis. However, limitation of this meta-analysis should also be noted. First, all data analyzed were directly extracted from original articles, and the data reported by each study differed significantly. No sufficient data were available to perform further stratified analyses, such as smoking, gender, chemotherapy regimens, performance status and follow up. Second, the number of studies included in each subgroup was small, especially for sub-group analysis of XPD polymorphisms and OS. Third, toxicity is an important issue of Pt-chemotherapy. Due to the fact that few studies have reported the correlation between XPD polymorphisms and toxicity, we did not analyze toxicity profiles.

To summary, in the present meta-analysis we found that XPD Lys751Gln polymorphism was not associated with clinical outcomes of Pt-chemotherapy in NSCLC, while the XPD Asp312Asn polymorphism could predict poor response to Pt-chemotherapy, favorable survival in Caucasian but unfavorable survival in Asian population. Further studies with large sample size are warranted to validate these conclusions.

## Supporting Information

Table S1 **PRISMA checklist.**
(DOC)

Table S2 **Quality assessment of eligible studies with Newcastle-Ottawa Scale.**
(DOCX)
