# Supplementary material for: Predictive Value of XPD Polymorphisms on Platinum-Based Chemotherapy in Non-Small Cell Lung Cancer: A Systematic Review and Meta-Analysis
Source: PLoS One. 2013 Aug 19;8(8):e72251. doi: 10.1371/journal.pone.0072251 (PMC3747109; doi:10.1371/journal.pone.0072251)
Supplement: Table S2 — Quality assessment of eligible studies with Newcastle-Ottawa Scale. (DOCX) [file pone.0072251.s002.docx]

Table S1. Table S1. Quality assessment of eligible studies with Newcastle-Ottawa Scale

| Author | Year | Selection | Comparability | Outcome |
| --- | --- | --- | --- | --- |
| Provencio M | 2012 | ☆☆☆☆ | ☆☆ | ☆☆ |
| Zhang ZY | 2012 | ☆☆☆ | ☆☆ | ☆☆☆ |
| Tiseo M | 2012 | ☆☆☆☆ | ☆☆ | ☆☆ |
| Liao WY | 2012 | ☆☆☆☆ | ☆☆ | ☆☆☆ |
| Wu W | 2012 | ☆☆☆☆ | ☆ | ☆☆☆ |
| Chen X | 2012 | ☆☆☆☆ | ☆ | ☆☆☆ |
| Ludovini V | 2011 | ☆☆☆☆ | ☆☆ | ☆☆☆ |
| Joerger M | 2012 | ☆☆☆☆ | ☆☆ | ☆☆☆ |
| Li D | 2012 | ☆☆☆☆ | ☆ | ☆☆☆ |
| Ren S | 2012 | ☆☆☆☆ | ☆ | ☆☆☆ |
| Liu L | 2011 | ☆☆☆☆ | ☆ | ☆☆☆ |
| Viñolas N | 2011 | ☆☆☆☆ | ☆☆ | ☆☆☆ |
| Li F | 2010 | ☆☆☆☆ | ☆ | ☆☆☆ |
| Yao CY | 2009 | ☆☆☆☆ | ☆ | ☆☆ |
| Gandara DR | 2009 | ☆☆☆☆ | ☆☆ | ☆☆☆ |
| Kalikaki A | 2009 | ☆☆☆☆ | ☆ | ☆☆☆ |
| Tibaldi C | 2008 | ☆☆☆☆ | ☆☆ | ☆☆☆ |
| Yuan P | 2006 | ☆☆☆☆ | ☆ | ☆☆☆ |
| de las Peñas R | 2006 | ☆☆☆☆ | ☆☆ | ☆☆ |
| Isla D | 2004 | ☆☆☆☆ | ☆☆ | ☆☆☆ |
| Gurubhagavatula S | 2004 | ☆☆☆☆ | ☆ | ☆☆☆ |
| Ryu JS | 2004 | ☆☆☆☆ | ☆☆ | ☆☆☆ |
